# Assessments of *Vibrio parahaemolyticus* and *Vibrio vulnificus* levels and microbial community compositions in blue crabs (*Callinectes sapidus*) and seawater harvested from the Maryland Coastal Bays

**DOI:** 10.3389/fmicb.2023.1235070

**Published:** 2023-09-28

**Authors:** Jasmine Smalls, Christopher Grim, Salina Parveen

**Affiliations:** ^1^Department of Agriculture, Food and Resource Sciences, Food and Agricultural Sciences Program, University of Maryland Eastern Shore, Princess Anne, MD, United States; ^2^Center for Food Safety and Applied Nutrition, U.S. Food and Drug Administration, College Park, MD, United States

**Keywords:** crabs, *Vibrio parahaemolyticus*, *Vibrio vulnificus*, microbial community, physicochemical parameters

## Abstract

**Introduction:**

Fluctuations in environmental physicochemical parameters can affect the diversity and prevalence of microbial communities, including vibrios, associated with aquatic species and their surrounding environments. This study aimed to investigate the population dynamics of two *Vibrio* species as well as the microbial community diversity of whole crab and seawater from the Maryland Coastal Bays (MCBs), using 16S rRNA sequencing.

**Methods:**

During this study, three crabs and 1 L of seawater were collected monthly from two sites for 3 months. Crab tissue was extracted and pooled for each site. Extracted crab tissue and seawater were analyzed for *Vibrio parahaemolyticus* and *V. vulnificus* using Most Probable Number (MPN) real-time PCR. For 16S rRNA microbiome analysis, three different DNA extraction kits were evaluated to extract microbial DNA from individual crabs. Also, 500 mL of each seawater sample was filtered for DNA extraction.

**Results:**

Results indicated that sample types and sampling periods had a significant effect on the alpha diversity of the microbial community of crabs and seawater (*p* < 0.05); however, no statistical difference was found between DNA extraction kits. Beta diversity analysis also found that the microbial compositions between sample types and temporal distributions were statistically significant. Taxonomic classification revealed that Proteobacteria, Cyanobacteria, Actinobacteria, and Bacteroidetes were present in both crab and seawater samples. *Vibrio parahaemolyticus* and *V. vulnificus* were also detected in both crab and seawater samples, although crabs contained a higher concentration of the bacterium compared to the seawater samples. It was found that vibrios were not a dominant species in the microbial community of crab or seawater samples.

**Discussion:**

Results from this study provide further insight into species diversity and phylogenetic compositions of blue crabs and seawater from the MCBs. These approaches will help in risk assessments that are essential in the overall advancement of public health.

## Introduction

*Vibrio* spp., in which some can cause human infections, exists naturally in marine and estuarine environments. *Vibrio*-related illnesses can occur by consumption of contaminated, undercooked, or raw shellfish, primarily oysters, or exposure of an open flesh wound to water containing high levels of pathogenic vibrios (Altekruse et al., 2000; Baker-Austin et al., 2018). *Vibrio* infections are more likely to increase during summer months as the increase in water temperatures favors the proliferation of these species. Johnson et al. (2012) found temperature to be a reliable predictor of the prevalence and dispersal of *Vibrio* spp. when investigating the relationship between environmental factors and *Vibrio* spp. in oysters, sediment, and water across a multi-coastal region. Rodgers et al. (2014) also reported that *Vibrio* levels were positively correlated to temperature, with concentrations being most abundant during warmer months. Moreover, correlations between bacteria such as *Vibrio* spp. and physicochemical parameters, such as temperature and salinity, can influence the microbial composition in crabs and seawater (Deng et al., 2020).

Increasingly, the interaction between pathogens and microbiome populations, such as *Vibrio* spp. and the crab microbiome, has become an area of great interest. A microbiome is a population or community of microorganisms that consist of bacteria, fungi, archaea, viruses, and other microbes associated with an ecological niche or that reside on or in various regions of a host (Nayak, 2010; Shreiner et al., 2015). Determining the composition of a microbial community can be done using metagenomics, a sequencing-based approach that allows for the complete microbiota to be characterized (Martínez-Porchas and Vargas-Albores, 2017). Although pathogens and other bacterial species associated with crabs have been investigated (Wang, 2011), research examining the microbial community of blue crabs in their entirety is limited.

Givens et al. (2013) described the population structure of the microbiota of hemolymph, gut, and carapace (shell) of blue crabs, by sequencing 16S rRNA amplicon clone libraries. The authors reported that the microbiome of blue crabs was diverse with a high abundance of Proteobacteria, dominated by *Escherichia* spp., *Alteromonas* spp., and *Vibrio* spp., found in all crab sample types examined. It was noted, however, that the abundance of each microorganism varied among crab host sites, with each sample type having different taxonomic profiles. Of the sampled regions, the microbiota obtained from the gut samples were the most diverse community. Similarly, Ramachandran et al. (2018) also reported Proteobacteria to be the dominant phylum across all crab anatomical sites tested. However, this study found that crab meat and claws contained the most diverse microbial community, in contrast to the gut microflora reported by Givens et al. (2013). Wang et al. (2019) investigated the intestinal microbiome of Chinese mitten crabs and identified four core phyla, Proteobacteria, Tenericutes, Bacteroidetes, and Firmicutes.

The detection of various bacterial pathogens has also been observed in seawater. Seawater serves as an ecological niche for countless microbes (Baker-Austin et al., 2018; Letchumanan et al., 2019). Abrupt or abnormal changes in water physicochemical parameters can adversely influence the microbial communities of aquatic organisms (Hillebrand et al., 2018). When comparing the microbial communities of crabs and seawater, it has been reported that each has a distinct microbiome. Although, certain bacteria are found within both microbial communities, a previous study have reported that seawater contains a more diverse microbiome than crabs (Sun et al., 2020a). The microbiota is of immense importance because it can serve as a defensive barrier against pathogens. Changes in environmental conditions can cause opportunistic bacterial pathogens such as *Vibrio* spp. to increase in number and accumulate in tissues and hemolymph of shellfish (Destoumieux-Garzón et al., 2020). However, there have been no studies to date that have investigated the levels of *Vibrio* and the microbial community in crabs and their surrounding environment simultaneously to examine the impact that *Vibrio* spp. has on the overall diversity of the microbiome.

The blue crab is economically important (Ramachandran et al., 2018; Waddell et al., 2020) and has been reported to contain pathogenic bacteria (Rodgers et al., 2014). Therefore, the purpose of this study was to determine (1) the prevalence of total *Vibrio parahaemolyticus* (*tlh^+^*) and *V. vulnificus* (*vvhA^+^*), as well as pathogenic *Vibrio parahaemolyticus* (*tdh^+^* and *trh^+^*) and clinical *V. vulnificus* (*vcgC^+^*-type) in blue crabs and seawater, (2) the correlation between *Vibrio* levels and selected physicochemical parameters (i.e., temperature, pH, dissolved oxygen, and salinity), and examine (3) the microbial composition of whole blue crabs and surrounding seawater from the Maryland Coastal Bays.

## Materials and methods

### Study area

**Figure 1:**
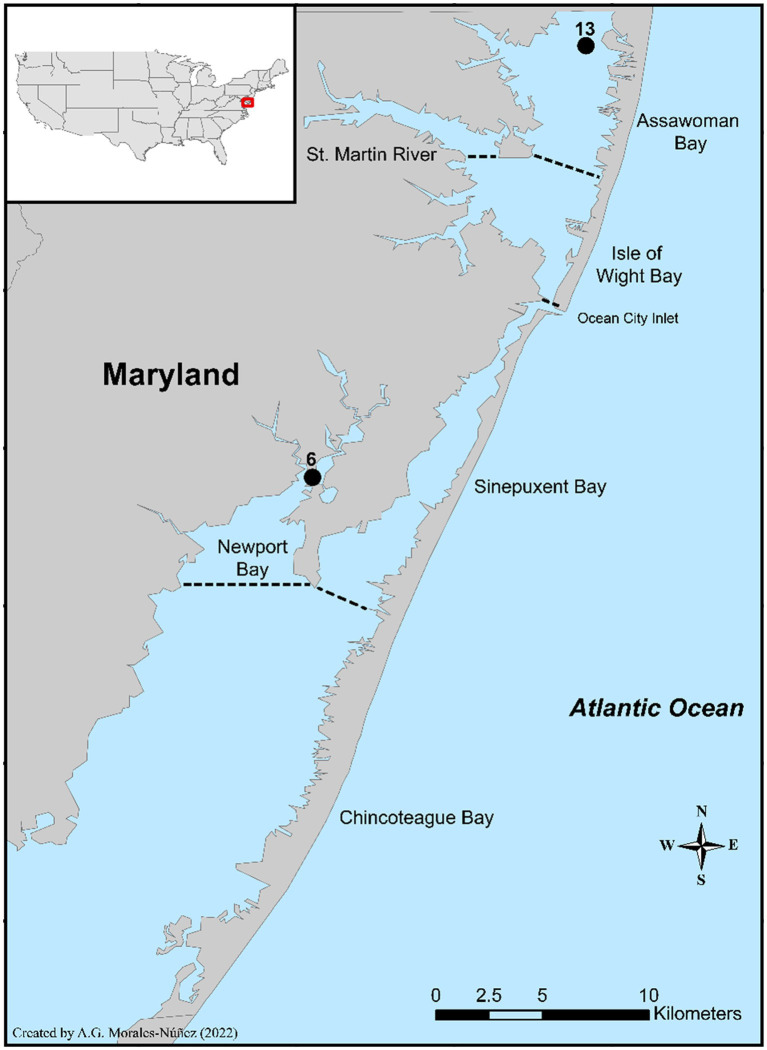
Location of the two sampling sites in the Maryland Coastal Bays.

A three-month study was conducted to gain a better understanding of *V. parahaemolyticus* and *V. vulnificus* abundances and the total microbiome diversity of blue crabs and their surrounding aquatic environment. For this study, blue crabs and seawater samples were collected from two sites, site 6 (New Port Bay) and site 13 (Assawoman Bay) within the Maryland Coastal Bays (MCBs; Figure 1). Site 6 is in the south region and positioned at the mouth of the bay. There is a golf course and a farm located near this site. Due to the periodic use of pesticides and other organic materials from these establishments, the integrity of the water quality is impacted at this site, with an anticipated effect on the water microbiome. Site 13 is located farther to the north. Because this site is at the Delaware-Maryland border where there is constant land development and it is close to Ocean City, MD, a resort destination, the degradation of the water quality at this site is attributed to excess nutrient runoff caused by human impact. These sampling sites were selected based on salinity, the historical prevalence of *Vibrio* spp., accessibility, and availability of crabs.

### Sample collection

A total of 24 samples (18 crabs and 6 seawater samples) were collected from the two sites from July through September 2020. From each sampling site, at each sampling event (month), 3 crabs (7–14 cm) and 1 L of surrounding surface seawater were collected. Live crabs were collected using an otter trawl net, where the net was cast and towed along the bottom of the bay at 2.5 knots for 6 min. Water samples were collected just below the surface using a Masterflex water sampler system and transferred to a sterile 1 L polyethylene bottle. After collection, crabs were bagged and chilled in an insulated chest with an ice pack and covered with a sheet of bubble wrap to prevent direct contact of crabs with the ice pack and water. The temperature was monitored using a data logger to verify that the temperature was less than 10°C during the transportation of the live crabs. Based on preliminary observational data, there was minimal to no variation between surface and bottom water parameter values (<0.2). Therefore, during the collection of samples, seawater temperature, salinity, dissolved oxygen, and pH were measured 2 feet below the surface using a portable multiparameter meter (YSI, Yellow Springs, OH, United States). Both water and crab samples were processed within 24 h of collection using Food and Drug Administration Bacteriological Analytical Manual (FDA-BAM) protocols, detailed below.

### Sample preparation

#### Crab meat extraction

Each sample, which was comprised of one crab, was scrubbed and dissected. The whole crab homogenate (meat and liquor) was weighed and transferred to a laboratory blender. A 1:1 dilution of the crab homogenate with sterile phosphate-buffered saline (PBS; w/v) was made and blended at maximum speed for 90 s using a sterile Waring blender jar (Waring, Stamford, CT, United States). After blending, a three-tube Most Probable Number (MPN) was conducted, where 2 mL of the homogenized sample was added to three 8 mL tubes of Alkaline Peptone Water (APW) and one tube 8 mL containing of PBS to obtain a 10^−1^ shellfish: diluent homogenate. Subsequently, an additional 1 mL of the 10^−1^ dilution was transferred and inoculated into an additional 9 mL of PBS to obtain a 10^−2^ dilution. This was done until a 10-fold serial dilution of 10^−8^ was obtained. From each tube, 1 mL of the diluent was transferred and inoculated into three 9 mL tubes of APW for each dilution. A portion of the remaining shellfish homogenate was aliquoted into two 1.5 mL centrifuge tubes and frozen at -80°C for DNA extraction.

DNA extractions were done within 48 h. using three different DNA Extraction Kits: (1) DNeasy PowerSoil Pro, (2) DNeasy Blood and Tissue (Qiagen, Germantown, MD, United States), and (3) ZymoBIOMICS Mini-Prep Kit (Zymo Research Corporation, Irvine, CA, United States) following the manufacturer’s instructions. A microbial community standard (ZYMO Research Corporation, Irvine, CA, United States) was also used to validate the efficiency of each DNA isolation protocol and relative abundance values were compared to the manufacturer’s theoretical values.

#### Seawater processing

For enumeration of *V. parahaemolyticus* and *V. vulnificus*, a three-tube MPN was conducted, where 10 mL of seawater was first inoculated into 100 mL of APW. Then, 1 mL of seawater was added to three tubes of 9 mL of APW and one 9 mL tube of PBS to obtain a 10^−1^ dilution. Serial dilutions and MPN methods were conducted using the procedure stated in the previous section. In addition, 500 mL of seawater was filtered using a 0.22 μm Sterivex filter (Manufacturer, City, State). The inoculated filters were then stored at −80°C until thawed for DNA extraction. DNA extractions were done using a DNeasy Power Water Extraction Kit (Qiagen, Germantown, MD, United States), following the manufacturer’s instructions.

#### PCR analysis

Presumptive *Vibrio* cultures, obtained from positive MPN tubes, were confirmed using real-time polymerase chain reaction (qPCR) assays to analyze five target genes during this study. A multiplex qPCR assay with an Internal Amplification Control (IAC) was used to confirm total (*tlh^+^*) and pathogenic (*tdh^+^* and *trh^+^*) *V. parahaemolyticus* (Nordstrom et al., 2007). A single-plex qPCR assay with an IAC was used to confirm total (*vvhA^+^*) *V. vunlificus* (Panicker et al., 2004), and clinical (*vcgC^+^*-type) *V. vulnificus* (Baker-Austin et al., 2010). qPCR reactions were prepared in a 25 μL reaction volume. All reactions were performed using an ABI 7500 Real-Time PCR system (Applied Biosystems, Foster City, CA). For the detection of species-specific genes (*tlh^+^* and *vvhA^+^*), samples were confirmed to be positive if samples amplified within 35 cycles, while 40 cycles were the threshold for pathogenic/clinical genes (*tdh^+^*, *trh^+^*, and *vcgC^+^*-type; Nordstrom et al., 2007; Baker-Austin et al., 2010).

### 16S rRNA microbiome profiling

A two-step amplification protocol was utilized for 16S rRNA amplicon sequencing library preparation of metagenomic DNA from crabs and seawater. This two-step amplification protocol was modeled after the published Illumina protocol (Illumina 16S Metagenomic Sequencing Library Preparation; Amplicon et al., 2013), with minor modifications. The V4 to V5 hypervariable regions of the 16S rRNA gene of each metagenome were first amplified using the forward primer, 515F (5′ TCG GCA GCG TCA GAT GTG TAT AAG AGA CAG GTG YCA GCM GCC GCG GTA 3′), and reverse primer, 926R (5′ GTC TCG TGG GCT CGG AGA TGT GTA TAA GAG ACA GCC GYC AAT TYM TTT RAG TTT 3′), as reported by Parada et al. (2016). Omni Klentaq and a PCR enhancer cocktail, PEC-1 (DNA Polymerase Technology, Inc., St. Louis, MO, United States) were utilized. Each 25 μL reaction consisted of 4.5 μL 5× Omni Klentaq Buffer, 0.5 μL Omni Klentaq Polymerase, 10.0 μL PCR Enhancer Cocktail, 1.5 μL each of 515F and 926R (600 nM final concentration), 2 μL nuclease-free water, and 5 μL of template DNA (1–5 ng/μL). There were 25 cycles of PCR amplification (40 s at 94°C, 30 s at 56°C, 45 s at 68°C) with an initial 2-min hot start at 94°C and a final extension step (5 min at 68°C). Agencourt AMPure XP beads (Beckman Coulter, Brea, CA, United States) were used to purify PCR products at 0.8× sample volume.

In the second round of PCR, Illumina MiSeq compatible Nextera XT indexes (Illumina Inc., San Diego, CA) were added to 16S rRNA library amplicons using a reduced cycle PCR protocol. Each 50 μL PCR reaction consisted of 9.75 μL 5× Omni Klentaq Buffer, 0.25 μL Omni Polymerase, 25 μL nuclease-free water, and 5 μL each of Illumina Nextera XT i5 and i7 indexing primers, in a unique combination, and 5 μL of purified library amplicon from the first PCR reaction. The limited cycle PCR amplification consisted of a 3 min hot start at 95°C, followed by 8 cycles of 30 s at 95°C, 30 s at 55°C, 30 s at 72°C, and a finishing extension step (5 min at 72°C). Agencourt AMPure XP beads were used to size select and purify libraries utilizing a 1.12× sample volume. Final libraries were quantified using the Qubit 3.0 fluorometer (Life Technologies, Carlsbad, CA, United States), and the libraries were pooled equimolarly at 4 nM. Library pools were denatured with 0.2 N NaOH, diluted to 8 pM with Illumina HT1 buffer, spiked with 15% 8 pM PhiX Control v3 (Illumina Inc., San Diego, CA, United States), and sequenced on a MiSeq benchtop sequencer using the MiSeq Reagent v3 600-cycle (2× 300 bp paired-end) kit (Illumina Inc., San Diego, CA). The 16S MTP (Microbiome Taxonomic Profiles) pipeline was used to analyze sequencing reads on the EZBioCloud Platform (EZBiome, Inc., Gaithersburg, MD).

### Statistical analysis

Raw data was archived in MS Excel. MPN levels (Log10 g^−1^ or mL^−1^) were determined using the FDA-BAM MPN calculation database. *Vibrio* log MPN values for crabs were further categorized into four classes (low (1–2 log g^−1^), lower middle (2–3 log g^−1^), upper middle (3–4 log g^−1^), and high (4–5 log g^−1^) for *V. parahaemolyticus* and undetected (<0 log g^−1^), low (0.1–1 log g^−1^), middle (1–2 log g^−1^), and high (2–3 log g^−1^) for *V. vulnificus*) to aid analysis of microbial distribution for enumerated *Vibrio* levels. *Vibrio* log MPN categorization was not conducted for seawater samples due to the low number of seawater samples examined.

A one-way Analysis of Variance (ANOVA) was used to determine statistically significant differences between concentrations of total and pathogenic/clinical vibrios among sites and sampling periods. A Spearman’s Rank Correlation analysis was used to assess if there is a correlation between concentrations of total (*tlh^+^* and *vvhA^+^*) and pathogenic/clinical (*tdh^+^, trh^+^*, and *vcgC^+^*-type) *Vibrio* spp. cultures and water quality parameters (temperature, salinity, dissolved oxygen, and pH). Due to the low occurrence of *Vibrio* spp. in seawater samples examined, 1 was added to all seawater MPNs to avoid and override negative log MPN concentrations.

A Wilcoxon Rank Sum test was used to determine if there were any statistically significant differences between microbial diversity among (1) DNA extraction kits, (2) sampling sites, (3) sampling periods, and (4) sample types. The assumptions that are necessary to be met while conducting these analyses were checked. Pretreatments on retrieved data were performed as needed. The homogeneity of variance was tested by using a Leven Test with a value of p of 0.05. Normality was checked using a Shapiro–Wilk test with a significance value of 0.05. A LEfSe Linear discriminant analysis (LDA) was used to determine the statistical significance of biological biomarkers between microbial compositions in crabs when analyzed according to *Vibrio* log MPN concentrations.

## Results

### Prevalence of total (*tlh^+^*) and pathogenic (*tdh*^+^ and *trh*^+^) *Vibrio parahaemolyticus* and total (*vvhA^+^*) and clinical (*vcgC^+^-*type) *Vibrio vulnificus*

**Figure 2:**
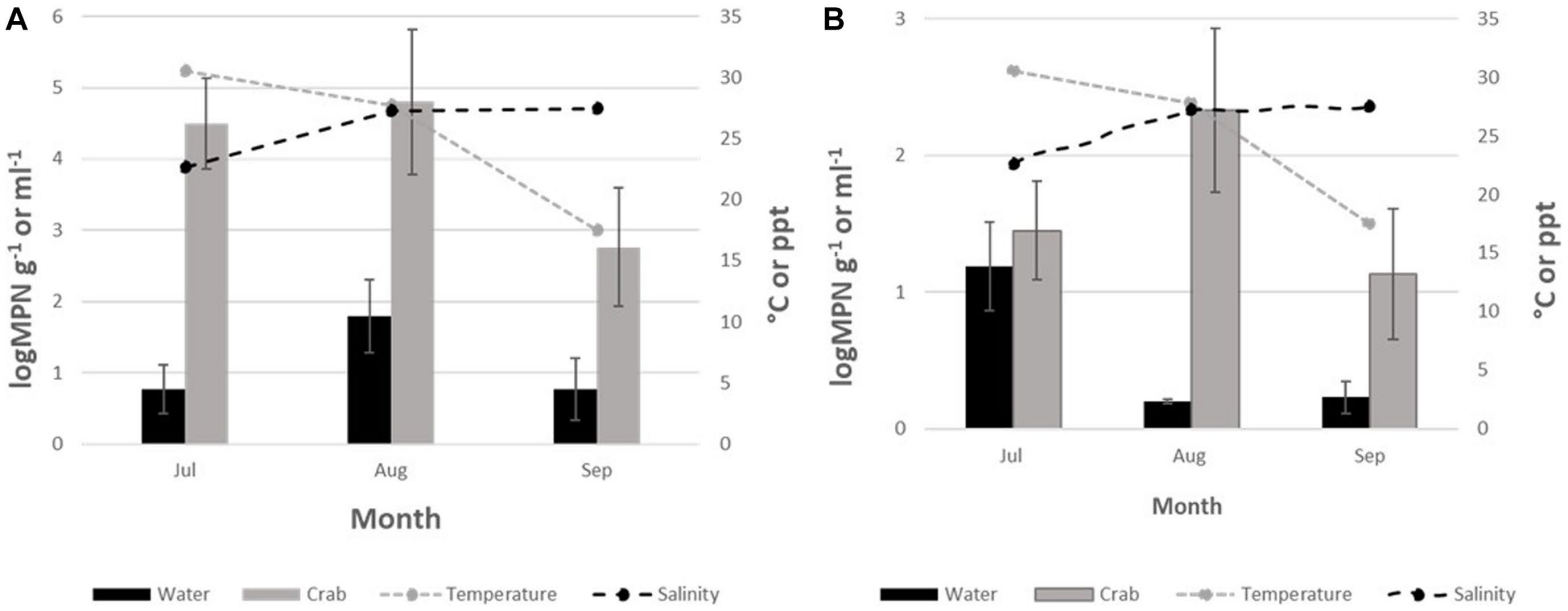
**(A)** Total *Vibrio parahaemolyticus (tlh+)* and **(B)** Total *Vibrio vulnificus (vvhA4+)* crabs and seawater from July to September 2020. The x-axis represents the months sampled. The left y-axis represents the average level of total Vibrio for two sites. The right y-axis represents the average seawater temperature and salinity levels.

*Vibrio parahaemolyticus* (*tlh^+^*) was present in all crab samples. Concentrations ranged from 2.7 to 4.8 log MPN g^−1^ (Figure 2A). Levels moderately increased between July and August before decreasing in September. Although, *V. parahaemolyticus* (*tlh*^+^) was detected in seawater samples (*n* = 6), concentrations were low at <2 log MPN mL^−1^ for the entire study and ranged from 0.7 to 1.8 log MPN mL^−1^. In water, a moderate increase in levels was observed from July to August before decreasing again in September (Figure 2A). There were no statistical differences found for the levels of this bacterium in either crab (*p* = 0.92 and *p* = 0.46) or seawater (*p* = 0.32 and *p* = 0.68) among sites or months. All crab and seawater samples examined had low frequencies of pathogenic *V. parahaemolyticus* (*tdh^+^* and *trh^+^*), therefore, MPN calculations and statistical analysis could not be conducted.

*Vibrio vulnificus* (*vvhA*^+^) was detected in all crab samples; however, the levels were low, with values below 2.5 log MPN g^−1^ for the entire study (Figure 2B). Concentrations ranged from 1.2 to 2.3 log MPN g^−1^. Levels were highest during July and gradually declined between August and September. Minimal detection of *V. vulnificus* (*vvhA*^+^) was observed in seawater cultures tested, 21.8% (39/179), with concentrations < 0.2 log MPN mL^−1^ for the entire study. Concentrations in seawater ranged from 0.2 to 1.3 log MPN mL^−1^. There was a moderate increase in levels during August before decreasing again in September (Figure 2B). There was no statistical difference found for the levels of this bacterium in either crab (*p* = 0.27 and *p* = 0.68) or seawater (*p* = 0.88 and *p* = 0.68) samples among sites or months. Due to low or undetectable occurrences of clinical *V. vulnificus* (*vcgC*^***+***^*-*type) in all samples, MPN calculations and statistical analysis could not be conducted.

### Relationship between physicochemical parameters, and total (*tlh^+^*) pathogenic (*tdh^+^*) and (*trh^+^*) *Vibrio parahaemolyticus*, and total (*vvhA^+^*) and clinical (*vcgC^+^*-type) *Vibrio vulnificus*

Environmental parameters observed during this study are outlined in Table 1. The seasonal mean of seawater temperatures observed were 25.67°C and 24.8°C at sites 6 and 13, respectively. The range of salinity levels at sites 6 and 13 was observed to be 21.93–27.93 and 23.32–28.44 parts per thousand (ppt), respectively. Dissolved oxygen (DO) levels were low, with values ranging from 4.55 (July, site 13) to 8.39 mg 1^−1^ (September, site 13). The pH values remained consistent throughout the study for both sites with a range of 7.36 (August) to 7.73 (July).

**Table 1:** Physicochemical parameters observed at two sites for 3 months.

Month	Site	Temperature (°C)	Sal (ppt)	DO (mg/L)	pH
July	6	30.7	21.93	6	7.73
	13	30.3	23.32	4.55	7.57
August	6	27.7	27.93	6.63	7.61
	13	27.8	26.61	5.21	7.36
September	6	18.6	26.49	6.52	7.52
	13	16.3	28.44	8.39	7.67

The relationships between *Vibrio* spp. and physicochemical parameters are summarized in Table 2. *Vibrio parahaemolyticus* (*tlh*^+^) in crabs did not show a significant relationship with temperature (*p* = 0.24), salinity (*p* = 0.56), DO (*p* = 0.18), or pH (*p* = 0.66). Likewise, *V. parahaemolyticus* (*tlh*^+^) in seawater also did not show a significant relationship with temperature (*p* = 0.42), salinity (*p* = 0.71), DO (*p* = 1), or pH (*p* = 0.92). As a result of the low occurrences, a significant correlation could not be determined between pathogenic *V. parahaemolyticus* (*tdh*^+^ and *trh*^+^) in either crab or seawater samples and physicochemical parameters.

**Table 2:** *Vibrio* spp. relationships with physicochemical parameters using Spearman’s rank correlation coefficient (rs).

Sample type	Gene target	*N*	Temperature	Salinity	DO	pH
Crab	*vvhA^+^*	18	0.54	−0.6	^*^−0.94	−0.66
	*vcgC^+^*	18	ND	ND	ND	ND
	*tlh^+^*	18	0.6	−0.31	−0.66	−0.26
	*tdh^+^*	18	ND	ND	ND	ND
	*trh^+^*	18	ND	ND	ND	ND
Seawater	*vvhA^+^*	6	−0.55	^*^0.81	0.29	−0.52
	*vcgC^+^*	6	ND	ND	ND	ND
	*tlh^+^*	6	0.43	−0.2	−0.029	0.086
	*tdh^+^*	6	ND	ND	ND	ND
	*trh^+^*	6	ND	ND	ND	ND

N represents the number of samples analyzed. ND indicates that statistical analyses were not determined due to low occurrences of the gene. ^*^Denotes significance at 0.05.

*Vibrio vulnificus* (*vvhA*^+^) in crabs showed a significant negative relationship with DO (*p* = 0.017), while *V. vulnificus* (*vvhA*^+^) in crabs did not show a significant relationship with temperature (*p* = 0.3), salinity (*p* = 0.24), or pH (*p* = 0.18). *Vibrio vulnificus* (*vvhA*^+^) in seawater showed a significant positive relationship with salinity (*p* = 0.05). However, *V. vulnificus* (*vvhA*^+^) in seawater did not show a significant relationship with temperature (*p* = 0.26), DO (*p* = 0.58), or pH (*p* = 0.29). Due to infrequent detection and low densities, a significant correlation could not be determined between clinical *V. vulnificus* (*vcgC*^+^-type) in crab or seawater samples and physicochemical parameters.

### Impact of choice of DNA extraction method on microbiome composition analysis of blue crabs and seawater

**Figure 3:**
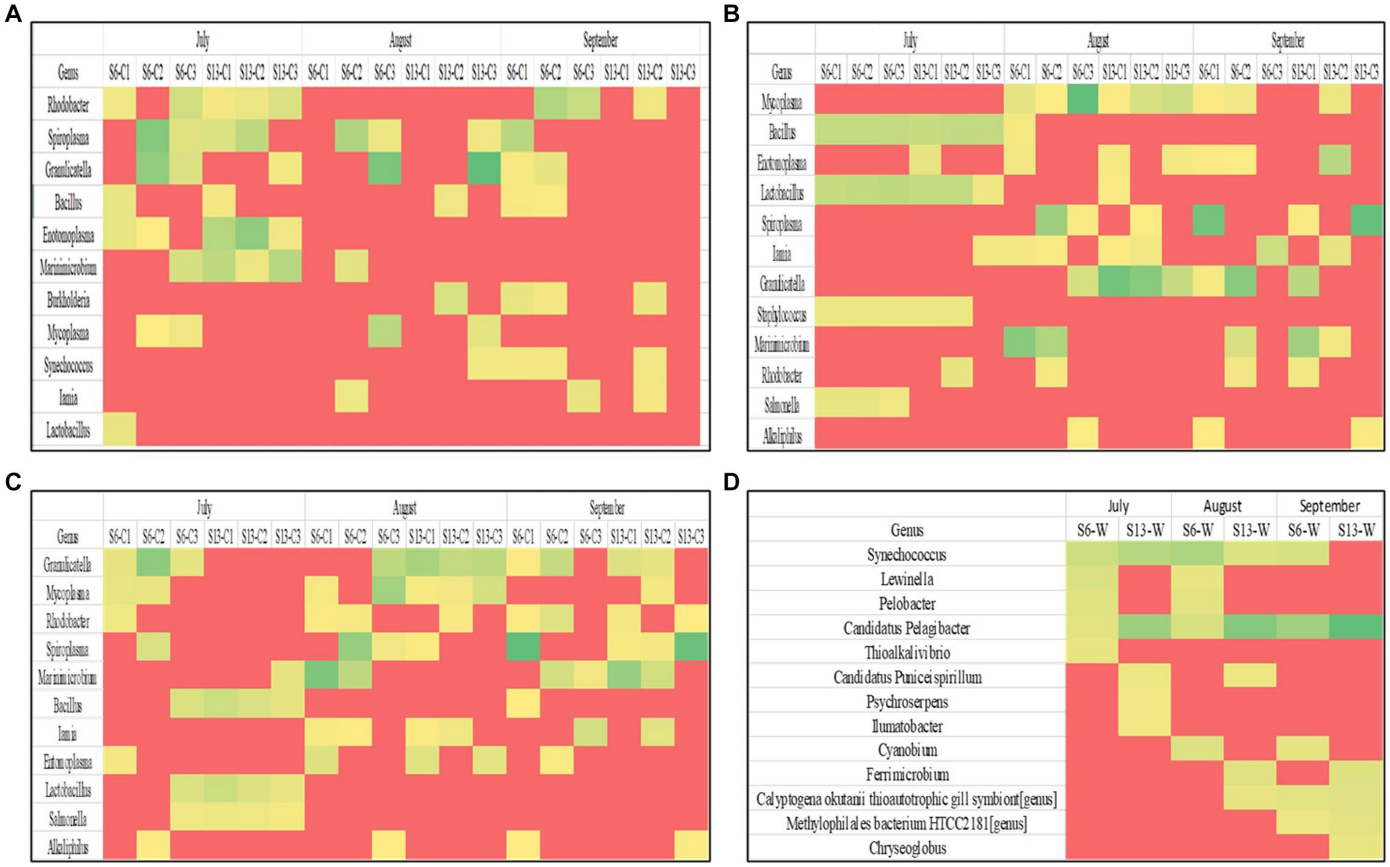
**(A–D)** Heat maps showing the dominant microorganisms in each sample grouped by DNA extraction kit. Each row represents a different genus within a sample. Each column represents an individual sample during a sampling period.

Taxonomic classifications from the 16S rRNA metabarcoding sequencing were analyzed in each crab and seawater sample to assess DNA extraction kit biases (Figures 3A–D). The microbial compositions varied between DNA extraction kits, sampling sites, and months. The metagenomic DNA extracted using the Blood and Tissue DNA extraction kit showed that the primary dominant genera for all crab samples were *Rhodobacter* and *Spiroplasma*, which were both detected in 44.4% of crab samples and were most prevalent in July. *Granulicatella, Bacillus*, *Enotomoplasma*, and *Marinimicrobium* were detected in 27.8 to 38.9% of crab samples and were primarily prevalent in July.

The metagenomic DNA that was isolated using the Pro Power Soil extraction kit indicated that the primary dominant genus present in all crab samples examined was Mycoplasma, which was detected in 50% of crab samples and was most prevalent in August. *Bacillus*, *Enotomoplasma*, *Lactobacillus*, *Iamia*, *Granulicatella*, and *Spiroplasm*a were detected in 33.3 to 38.9% of crab samples and were also primarily prevalent in August. The ZymoBIOMICS DNA Mini-Prep extraction kit yielded *Granulicatella* as the primary dominant genera for all crab samples, which was detected in 61.1% of crab samples and was most prevalent in August and September. *Mycoplasma*, *Rhodobacter*, *Spiroplasma*, and *Marinimicrobium* were detected in 38.9 to 44.1% of crab samples examined. Metagenomic DNA that was extracted from seawater samples using the Pro Power Water DNA extraction kit resulted in community profiles with *Candidatus Pelagibacter* as the primary dominant genus in all samples examined. *Synechococcus* was the second most abundant genus, as it was observed in 83.3% of samples.

A Wilcoxon-rank sum analysis found no statistically significant difference between the microbial community of crabs and the DNA Extraction kits utilized [Chao1 *p* = 0.849 (Blood and Tissue − Zymo), and 0.808 (Power Soil − Zymo); Shannon *p* = 0.323 (Blood and Tissue − Power Soil), 0.247 (Blood and Tissue − Zymo), 0.927 (Power Soil − Zymo); Simpson *p* = 0.544 (Blood and Tissue − Power Soil), 0.347 (Blood and Tissue − Zymo), 0.8769 (Power Soil − Zymo)]. A PERMANOVA also found no statistically significant difference in the distribution of the microbial composition of crabs between DNA Extraction kits (*p* = 0.575). Additionally, each method was tested using a microbial community standard (see Materials and Methods). The ZymoBIOMICS Mini-Prep Kit had the lowest deviation from expected values for the eight genera that comprised the community standard (Supplementary Figure 1), followed by the DNeasy PowerSoil Pro. The DNeasy Blood and Tissue kit had a substantial amount of bias in that it was inefficient in the extraction of DNA from cells of *Enterococcus faecalis* and *Listeria monocytogenes*.

### Microbial community compositions of blue crabs and seawater

**Figure 4:**
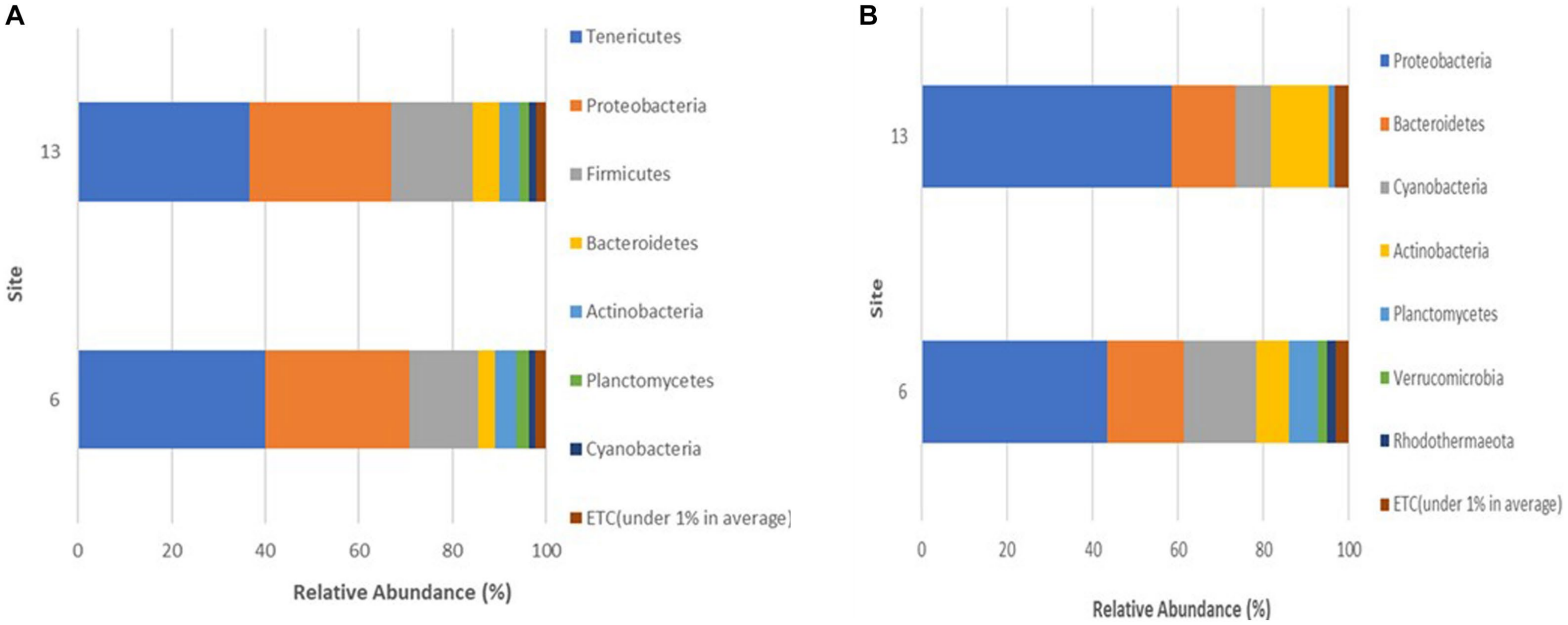
**(A,B)** Phyla detected in **(A)** crabs and **(B)** seawater harvested from two sites in the Maryland Coastal Bays.

The microbial community among crabs was observed to be highly diverse, with a total of seven different bacterial phyla detected in sequences retrieved from crab samples. The relative abundance for each phylum varied only slightly between sites during the study (Figure 4A), with Tenericutes as the most abundant phylum within the crab microbiome, accounting for 40.1% (Site 6) and 36.7% (Site 13) of the bacteria present. The second most abundant bacteria were those that belong to the phylum Proteobacteria, which accounted for 30% of the microbiota in crabs from both sites. Firmicutes accounted for 14.7% (Site 6) and 17.4% (Site 13) of the crab-associated microbial communities. Bacteria belonging to phyla Bacteroidetes, Actinobacteria, Planctomycetes, and Cyanobacteria had a relative abundance between 1 to 5% for both sites.

**Figure 5:**
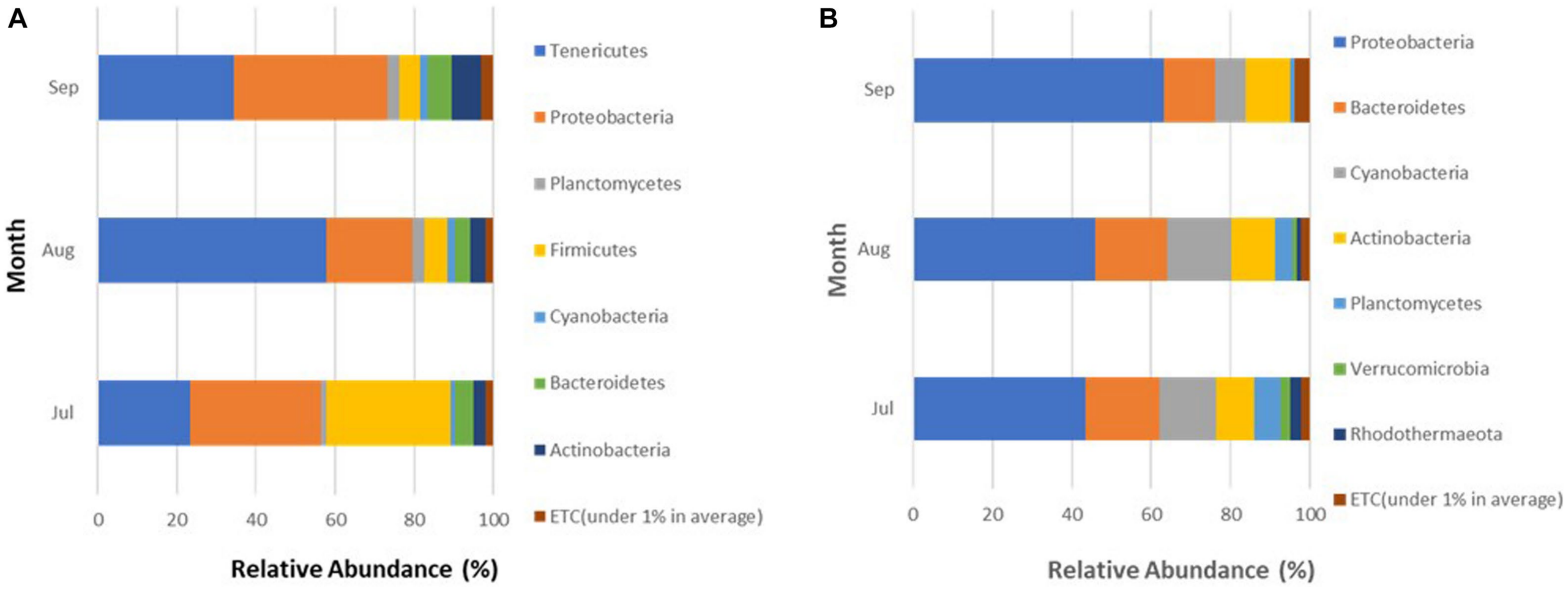
**(A,B)** Phyla detected in **(A)** crabs and **(B)** seawater harvested from Maryland Coastal Bays for 3 months.

Every month, the Proteobacteria phylum maintained a consistently high membership in the microbiome of crabs during all 3 months accounting for 33.2 (July), 22 (August), and 38.9% (September; Figure 5A). In contrast, members of the Tenericutes were more abundant in August (57.7%), compared to July (23.3%) and September (34.4%), and the Firmicutes spiked in July representing 31.4% of the microbial community in crabs.

A Wilcoxon-rank sum analysis found no statistically significant difference in the microbial community of crabs between the two sites (Chao1 *p* = 0.587; Shannon *p* = 0.162; Simpson *p* = 0.091). Significant differences in microbial community diversity were found, but these were also influenced by the diversity metric used. For example, there was a statistically significant difference in the microbial community of crabs between the of months August and September (Shannon *p* = 0.012) and July and August (Simpson *p* = 0.034). There was no statistically significant difference found by the Chao 1 index between July and August (*p* = 0.327), July and September (*p* = 0.297), August and September (*p* = 0.706); the Shannon index between July and August (*p* = 0.15), July and September (*p* = 0.54); and the Simpson index between July and September (*p* = 0.462), August and September (*p* = 0.083). Similarly, a PERMANOVA analysis (Jensen-Shannon) found no statistically significant difference in the distribution of the microbial composition in crabs between the two sites (p = 0.16), but a statistically significant difference in the distribution of the microbial composition of crabs between all months (*p* = 0.001).

A total of seven different phyla were detected in bacterial sequences retrieved from seawater samples (Figure 4B), but these were different from the phyla found in crab samples. The relative abundance for each taxon varied between sites, and the variation was larger than observed for the crab microbiome samples. Proteobacteria was the most abundant phylum in seawater, accounting for 43.4% (Site 6) and 58.5% (Site 13) of the bacteria present. The second most encountered bacteria were those that belong to the phylum Bacteroidetes, which accounted for 17.9% (Site 6) and 15% (Site 13) of the microbiota in seawater from both sites. Cyanobacteria accounted for 17.1% (Site 6) and 8.3% (Site 13) of the seawater microbial community. Actinobacteria also contributed to 7.6% (Site 6) and 13.5% (Site 13) of the bacteria present. Bacteria belonging to phyla Planctomycetes, Verrucomicrobia, and Rhodothermaeota all had a relative abundance between 1 to 6%, for both sites. Verrucomicrobia and Rhodothermaeota were only detected in the microbial community in seawater at site 6. Of the seven different phyla detected, the Proteobacteria phylum maintained a high concentration in the microbiome of seawater during all 3 months accounting for 43.4% (July), 46% (August), and 63.3% (September), gradually increasing during this study (Figure 5B). As Proteobacteria relative abundance increased from July to September, three of the phyla, Planctomycetes, Verrucomicrobia, and Rhodothermaeota seemingly decreased in an inverse relationship, while Cyanobacteria peaked in August (16%).

A Wilcoxon-rank sum analysis found no statistically significant difference in the microbial community of seawater between the two sites (Chao1 *p* = 0.127; Shannon *p* = 0.052; Simpson *p* = 0.127), or between months (Chao1 *p* = 1.000; Shannon *p* = 0.49; Simpson *p* = 0.333). Likewise, a PERMANOVA analysis (Jensen-Shannon) found no statistically significant difference in the distribution of the microbial composition of seawater between the two sites (*p* = 0.101) or between months (*p* = 0.465).

**Figure 6:**
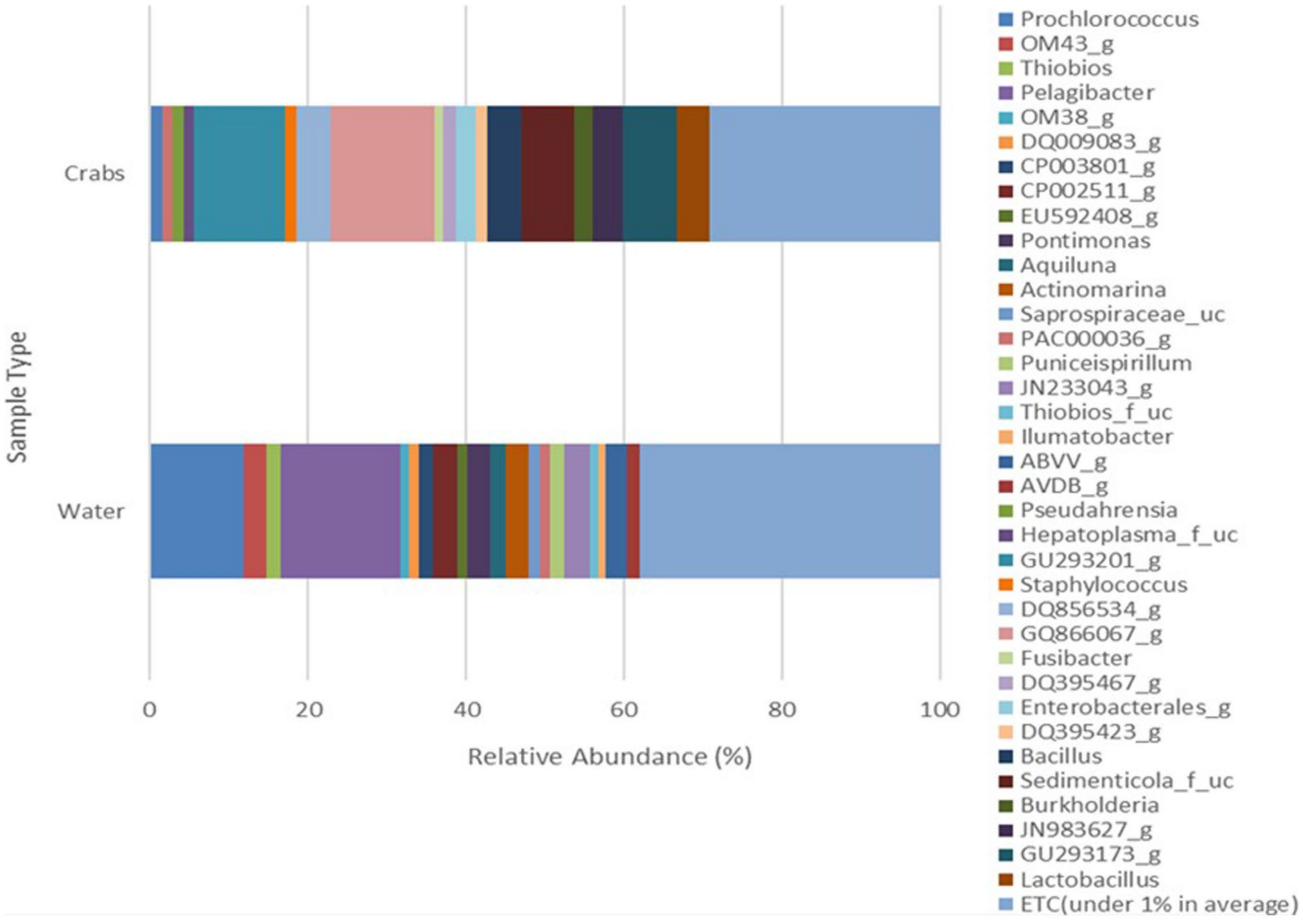
Genus microbial composition comparison of bacteria detected in crabs and seawater samples harvested from the Maryland Coastal Bays for 3 months.

**Figure 7:**
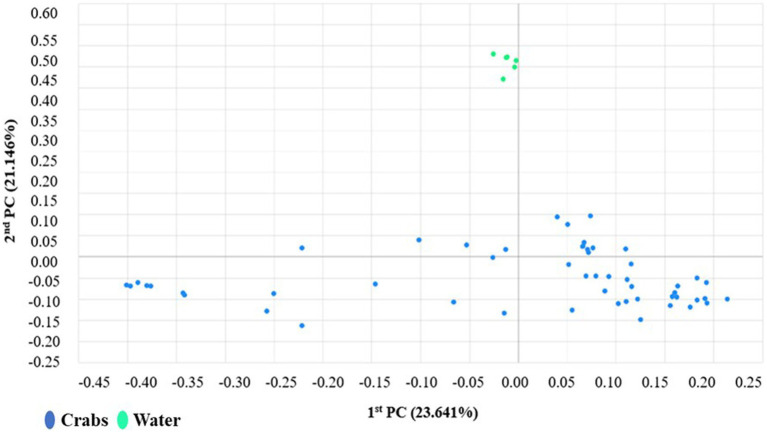
Coordinates scaling analysis of the distribution patterns of bacteria phyla found in carb and seawater samples harvested from the Maryland Coastal Bays.

The microbial composition of crabs varied from that of seawater (Figure 6). It was expected and observed that some genera were present in each microbiome, while niche-specific members were also observed. For instance, Proteobacteria, Bacteroidetes, and Actinobacteria were among the shared taxa between crabs and seawater. Tenericutes and Firmicutes were observed to be unique taxa specifically for crabs, whereas Verrucomicrobia and Rhodothermaeota were specific to only the seawater microbiome. As expected, the ordination of the genus-level taxonomic compositions of microbes for crab and seawater samples yielded niche-specific clustering (Figure 7). All crab samples clustered together and shared at least 65% similarity. All seawater samples clustered together and shared 100% similarity. A Wilcoxon-rank sum analysis found a statistically significant difference in the microbial community of sample type between crabs and seawater (Chao1 *p* = 0.001; Shannon *p* = 0.001; Simpson *p* = 0.001). A PERMANOVA (Jensen-Shannon) found a statistically significant difference in the distribution of the microbial composition of sample type between crab and seawater (*p* = 0.001).

### Microbial composition according to *Vibrio* spp. abundances

Crab samples were organized into four categories (see Materials and Methods) based on log MPN levels of *V. parahaemolyticus* and *V. vulnificus*, to identify potential synergistic and antagonistic cohort species in the crab microbiomes and potential interactions. The composite microbiomes of those categorized samples revealed distinctive microbiome profiles (Supplementary Figures 2A,B). In terms of *V. parahaemolyticus* enumeration categories (Supplementary Figure 2A) members of the families Sedimenticola and Morganellaceae (*Proteus* and unclassified), and a novel genus, GU293201 (*Hepatoplasma* sp.) appear to be more prevalent when *V. parahaemolyticus* log MPN values were low (17.6% and 29.51%) and decreased when log MPN values increased (4.39% and 3.9%), with the Morganellaceae members reduced to non-detectable levels. Conversely, certain taxa, such as novel genera GQ866067 (Lumbricoplasmataceae) and GU293173 (Lumbricoplasmataceae), displayed an inverse trend, where microbial abundance increased with *V. parahaemolyticus* log MPN values. Overall, crab microbiomes had a higher level of Proteobacteria when *V. parahaemolyticus* abundance was low and the community shifted to a higher proportion of Firmicutes as *V. parahaemolyticus* abundance increased.

As a general trend, microbial compositions appeared more diverse as *V. parahaemolyticus* log MPN values increased, compared to the lowest category. Statistical tests of microbial diversity differences between categories, gave confounding results, with those tests that emphasize species richness giving more significant differences between populations. A Wilcoxon-rank sum analysis found no statistically significant difference in the microbial composition of crabs between samples with low *V. parahaemolyticus* log MPN concentrations and those categorized with lower middle (Shannon *p* = 0.176; Simpson *p* = 0.106), upper middle (Chao 1 = 0.165), and high (Shannon *p* = 0.127) log MPN levels. Conversely, there was a statistically significant difference in the microbial community of crabs between samples with low *V. parahaemolyticus* log MPN concentrations and those categorized with lower middle (Chao 1 *p* = 0.005), upper middle (Shannon *p* = 0.033; Simpson *p* = 0.004) and high (Chao 1 *p* = 0.007; Simpson *p* = 0.046) log MPN levels. Similarly, a PERMANOVA analysis (Jensen-Shannon) found a statistically significant difference in the distribution of the microbial composition in crabs between samples with low *V. parahaemolyticus* log MPN concentrations and those categorized with lower middle (*p* = 0.014), upper middle (*p* = 0.036), and high (*p* = 0.014) log MPN levels.

Several diversity indices can be profoundly biased by a single sample or a few samples that have an unusual distribution of taxa by relative abundance. To account for biological inconsistency and statistically identify biomarkers in each category, the linear discriminant analysis (LDA) effect size (LEfSe) method of analysis was performed as previously described (Chang et al., 2022). The LEfSe analysis for *V. parahaemolyticus* abundance categories detected several taxa that were potential biomarkers associated with an increase in *V. parahaemolyticus* abundance (Supplementary Table 1). As was seen with the composite community profiles presented in Supplementary Figure 1, there is a general trend of several members of the Firmicutes, such as *Bacillus*, *Staphylococcus*, *Lactobacillus*, and Mollicutes increasing in abundance as the *V. parahaemolyticus* abundance increases across categories. Several other taxa follow this trend, including *Procholorococcus*, *Escherichia*, *Pseudomonas*, and genera of the Lumbricoplasmataceae.

The log MPN categorization *V. vulnificus* ranged from undetected to high. The microbial compositions of these crab samples also varied between categories (Supplementary Figure 2B), as was seen for *V. parahaemolyticus*. Unlike *V. parahaemolyticus* categories, strong trends of increasing cohort relative abundances across the increasing classes of *V. vulnificus* abundances were not observed. Some taxa, such as novel genera s JN983627 (Heptoplasma) and GU293173 and GQ866067 (Lumbricoplasmataceae), displayed a slight trend, where microbial abundance increased with *V. vulnificus* log MPN concentrations. Several taxa displayed non-linear trends. For example, novel genus GU293201 (Heptoplasma) appeared to be most prevalent in crab samples with low to middle (17.25% and 20.3%) *V. vulnificus* log MPN values compared to samples with high log MPN values (2.71%). Also, notable unclassified genera from *Thiobios* and *Saprospiraceae* were only present with low concentrations (2.33% and 2.17%) in crab samples where *V. vulnificus* was undetected.

A Wilcoxon-rank sum analysis found no statistically significant difference in the microbial composition of crabs between samples with undetected *V. vulnificus* and those categorized with low (Chao 1 *p* = 0.748; Shannon *p* = 0.927; Simpson *p* = 0.963), middle (Chao 1 *p* = 0.065; Shannon *p* = 0.339; Simpson *p* = 1.00), and high (Chao 1 *p* = 0.55; Shannon *p* = 0.358; Simpson *p* = 0.060) *V. vulnificus* log MPN levels. Likewise, a PERMANOVA analysis (Jensen-Shannon) found no statistically significant difference in the distribution of the microbial composition of crabs with undetected *V. vulnificus* and those categorized with low (*p* = 0.22) and middle (*p* = 0.247) log MPN levels. However, a PERMANOVA analysis (Jensen-Shannon) found a statistically significant difference in the distribution of the microbial composition of crabs with undetected *V. vulnificus* and those categorized with high (*p* = 0.22) *V. vulnificus* log MPN levels.

The LEfSe analysis for *V. vulnificus* abundance categories was also performed. In general, there were fewer biomarkers found as compared to *V. parahaemolyticus* categories at the same effect size. As was seen with low *V. parahaemolyticus* abundant crab microbiomes, samples with non-detectable or low levels of *V. vulnificus* had a high proportion of Proteobacteria, specifically Gamma-proteobacteria (Supplementary Table 2). For the mid-level of *V. vulnificus* crab samples, several members of the Delta and Gamma-proteobacteria were enriched compared to non-detectable *V. vulnificus* samples, while Alpha-proteobacteria and Flavobacteria members displayed a significant reduction in abundance in high *V. vulnificus* crab microbiomes.

## Discussion

The microbial community of blue crabs and seawater can be an excellent indicator of determining the quality and safety of the animal and its environment, which is important given the increase in seafood consumption. Our findings revealed that bacterial pathogen abundance was significantly influenced by sample types and months. For example, MPN results indicated that *Vibrio* spp. were observed to be more prevalent, and at higher levels, in crab samples than seawater, with a peak in August. *Vibrio*-related infections cause an estimated 80,000 illnesses, 500 hospitalizations, and 100 deaths in the U.S. each year (CDC, 2020). Globally, the aquaculture industry experiences economic losses due to bacterial diseases, including those attributed to *Vibrio* spp., resulting in the loss of millions of dollars. Hence, it is important to have efficient disease management practices. This is the most current study to date to examine the levels of *V. vulnificus* and *V. parahaemolyticus* along with the microbiome of individual crabs and seawater from the Maryland Coastal Bays.

*Vibrio parahaemolyticus* (*tlh*^+^) was detected in higher concentrations than *V. vulnificus* (*vvhA*^+^) and was observed to be more prevalent in August when temperature and salinity levels were optimal for this bacterium to propagate. Rodgers et al. (2014) also observed higher concentrations of *V. parahaemolyticus* (*tlh*^+^) compared to *V. vulnificus* (*vvhA^+^*) in similar sample types. In this study, crab samples harvested during August were found to have higher counts of *V. parahaemolyticus* (*tlh*^+^). Rodgers et al. (2014) reported similar findings, where higher concentrations of *V. parahaemolyticus* (*tlh*^+^) were observed in crabs during July and September where concentrations ranged approached 5 log MPN g^−1^ for both months. The levels from both studies were comparable, having ranges of >3 to <5 log MPN g^−1^for *V. parahaemolyticus* (*tlh*^+^). The detection of *V. parahaemolyticus* (*tlh*^+^) was low in seawater samples examined during this study. Similarly, Rodgers et al. (2014) also reported infrequent detection of *V. parahaemolyticus* (*tlh*^+^) from seawater samples collected from July through September. The detection of pathogenic *V. parahaemolyticus* (*tdh*^+^ and *trh*^+^) was extremely low in both crab and seawater samples. These results are consistent with previous findings that reported low occurrences of pathogenic *V. parahaemolyticus* (*tdh*^+^ and *trh*^+^; Rodgers et al., 2014; Almuhaideb et al., 2020; Parveen et al., 2020; Ndraha and Hsiao, 2021).

During this study, *V. vulnificus* (*vvhA*^+^) was detected in low concentrations in both crab and seawater samples examined. Among all samples examined, *V. vulnificus* (*vvhA*^+^) was most prevalent in crabs harvested in August. A similar study also observed higher concentrations of *V. vulnificus* (*vvhA*^+^) in crabs collected from July through September (Rodgers et al., 2014), where concentrations ranged from >1 to >4 log MPN g^−1^. In this study, *V. vulnificus* (*vvhA*^+^) enumeration was consistently below 1 log MPN g^−1^, for all positive samples. Differences between the two studies may be due to the slight variation in environmental factors, such as temperature and salinity, observed between the two studies. This is to be expected given that the salinity concentrations in the bays are above optimum levels for this bacterium (Deeb et al., 2018). Clinical *V. vulnificus* (*vcgC*^+^-type) was infrequently detected in both crab and seawater samples. It has been previously reported that environmental (*vcgE*^+^ allele) type strains of *V. vulnificus* were found to be more prevalent in the environment in the United States compared to the Asian environment which showed a greater concentration of clinical (*vcgC*^+^ allele) type strains (Kim and Jeong, 2001; Chatzidaki-Livanis et al., 2006; Mahmud et al., 2010; Froelich, 2012; Rodgers et al., 2014). Our findings might support this finding, given the low densities of clinical *V. vulnificus* (*vcgC*^+^-type) observed during this study. However, further research is needed to verify this claim.

The correlation between *V. vulnificus* (*vvhA*^+^) levels in seawater and salinity has been contradictory. A significant positive correlation was found between *V. vulnificus* (*vvhA*^+^) in seawater and salinity. This agrees with a previous study that reported the level of *V. vulnificus* (*vvhA*^+^) in seawater was positively correlated with salinity (Randa et al., 2004). Conversel*y,* Parveen et al. (2020) did not detect a correlation between *V. vulnificus* (*vvhA*^+^) levels in seawater and salinity. King et al. (2021) reported that *V. vulnificus* (*vvhA*^+^) levels in seawater had a weak correlation with salinity. These combined findings indicate that the abundance of *V. vulnificus* (*vvhA*^+^) in seawater does not solely rely on salinity alone but on combined interactions with additional environmental factors such as temperature, which may attribute to the variations observed in previous studies. Although further research is needed to fully determine the correlation between *V. vulnificus* (*vvhA*^+^) levels in seawater and salinity. Moreover, dissolved oxygen (DO) has not been reported to be correlated with the density fluctuations of *V. vulnificus* in estuary environments (Pfeffer et al., 2003); however, a significant negative correlation was found between *V. vulnificus* (*vvhA*^+^) in crabs and DO during this study. Parveen et al. (2020) and Rodgers et al. (2014) observed similar findings. This is because excess nutrients and consequent heterotrophic bacterial activity in the water column have the potential to decrease DO levels (Carpenter et al., 1998; Rathore et al., 2016).

Crab samples were collected from two different geographical locations within the MCBs. In essence, each site has distinct nutrients and environmental conditions that can ultimately influence the microbiome of the crabs depending on food accessibility and the health of the crab. More specifically, nutrient sources found in aquatic environments or feeding status can determine the gut content or quality of crabs (Sun et al., 2020b). This may be attributed to the detection of various bacterial species found in crab samples when categorized based on *Vibrio* spp. log MPN levels. Furthermore, given that certain bacteria require specific conditions for proliferation, it can cause one group of bacteria to overpopulate and outcompete other bacteria when present in the same environment or host. For example, the species richness and evenness varied across the different *Vibrio* spp. concentrations. This indicates that certain conditions were more favorable for *Vibrio* spp. compared to other bacteria allowing them to be more prevalent in examined samples. The contrary could also be noted, in which certain conditions were more favorable for novel species compared to *Vibrio* spp. allowing them to be more prevalent in examined samples. However further research is needed to better understand the specific relationship between *Vibrio* concentrations and the abundance of other species and how such relationship affects the microbial structure of blue crabs.

Different DNA extraction methods can produce variability in measurements of species richness, evenness, and overall total microbial composition within a community. Three different DNA extraction methods were used during this study to identify the microbial community of blue crabs, in which each extraction kit produced different dominant species for the same sample. This is because, for example, the Blood and Tissue extraction method consists of a chemical cell lysis process, resulting in the detection of fewer taxa (Hermans et al., 2018). The Power Soil and Zymo extraction methods, however, contain a more robust mechanical lysis process that allows for the disruption of more difficult-to-lyse cells. Furthermore, although the differences observed were not statistically significant, it can render a false notion that extraction methods do not have any bearing on the overall microbial composition in an organism. All extraction kits yielded a higher abundance of Gram-negative bacteria than Gram-positive. Gram-positive bacteria contain a thicker and stronger peptidoglycan layer making them harder to lyse (Liu et al., 2018), which results in the limited detection of Gram-positive bacteria within a microbial community (Hermans et al., 2018). Even though each isolation kit resulted in a different dominant genus, DNA extraction methods did not have a significant effect on the alpha diversity of microbial communities in crabs and seawater.

Indices such as Chao1, Shannon, and Simpson were used to determine the bacterial diversity among samples. The Shannon index takes into consideration the different species types within a sample. For instance, for this study, the Shannon index suggests that the bacterial diversity was lower in August compared to September, which suggests that more bacteria can adapt to conditions and become more stable in late summer compared to early to mid-summer. This may be attributed to the statistically significant difference observed between the 2 months. Conversely, the Simpson index measures and gives more weight to species evenness, meaning that bacterial concentrations are the primary focus. The Simpson index for this study suggests that certain bacterial concentrations were higher in July compared to August where they were less abundant. This may be attributed to environmental conditions being optimal for certain bacteria allowing them to proliferate and overpopulate in July compared to August. Also, although it appears that bacterial concentrations were different between July and September, they were not significant.

The current study found the microbial community of blue crabs to be surprisingly diverse, as previously reported by Givens et al. (2013). Tenericutes and Proteobacteria were the most abundant phyla within the crab microbiota. These findings are consistent with results previously reported on the microbial community of blue crabs (Harris, 1993; Oxley et al., 2002; Li et al., 2012; Givens et al., 2013; Zhang et al., 2016). Tenericutes and Proteobacteria are known to occupy the hindgut and foregut of various crustaceans, including blue crabs (Ooi et al., 2017). This is because Tenericutes become enriched with niche expansion (Rungrassamee et al., 2013) and Proteobacteria are the first colonizers of marine surfaces (Dang and Lovell, 2016). It also appeared that the ratio and abundance of Tenericutes and Proteobacteria are influenced by developmental stages, which could determine their potential dominance in the alimentary canal (foregut, midgut, and hindgut; Ooi et al., 2017).

During this study, it was also found that based on the microbial genus composition, seawater has a more diverse microbiota than crabs. A study conducted by Zhang et al. (2016) reported similar findings when comparing the microbial communities of crabs and seawater. It has been reported that not all microorganisms that exist in the water column would be able to be ingested and survive within the intestinal tract of crabs, as well as other aquatic species, thereby, making the crab microbiome less diverse (Huang et al., 2018). Interestingly, it would be assumed that the microbiome of crabs would be more diverse than seawater given that crabs are benthic or bottom feeders. Blue crabs are known to inhabit various environments and forage on any available items to fulfill nutritional requirements (Hamida et al., 2019), which would lead to a potential increase in the diversity of the microbiome.

In conclusion, this study revealed that although *Vibrio* spp. has been reported to naturally exist in the microbiota of crab and seawater, they are not a dominant bacterium and exists in low levels in the microbial community of crab and seawater samples observed from the Maryland Coastal Bays. *Vibrio* concentrations were consistent with results from Rodgers et al., 2014, although samples were collected 10 years ago from the same bay at different sites as the current study. These results indicate that climate change did not significantly affect the concentrations of *Vibrio*. However, the previous study did not investigate microbial composition using a metagenomic approach.

Blue crabs and seawater both have distinctively diverse and unique microbial communities. Physicochemical parameters are known to affect the microbial diversity in crabs and seawater; however, more research is needed to gain a more coherent understanding of those effects. Due to the limited sampling period for this study, not enough temporal samples were collected to capture a complete variation in physical parameters and *Vibrio* spp. abundance. Therefore, an extended sampling period is suggested to gain a more accurate depiction of the correlation between physical parameters and *Vibrio* spp. abundance. Also, because this study depended solely on the use of 16 s rRNA sequencing, species-level classification could not be assigned. It is suggested that the microbiome can be influenced by not only habitational regions but developmental stages and gut regions. Thus, the long-term implications on the productivity of the wild blue crab population in the Maryland Coastal Bays need to be further investigated using shotgun metagenomics.

## Data Availability

The datasets presented in this study can be found in online repositories. The names of the repository/repositories and accession number(s) can be found in the article/Supplementary material.
