# Supplementary figures and images for: Assessments of Vibrio parahaemolyticus and Vibrio vulnificus levels and microbial community compositions in blue crabs (Callinectes sapidus) and seawater harvested from the Maryland Coastal Bays
Source: Front Microbiol. 2023 Sep 28;14:1235070. doi: 10.3389/fmicb.2023.1235070 (PMC10581026; doi:10.3389/fmicb.2023.1235070)

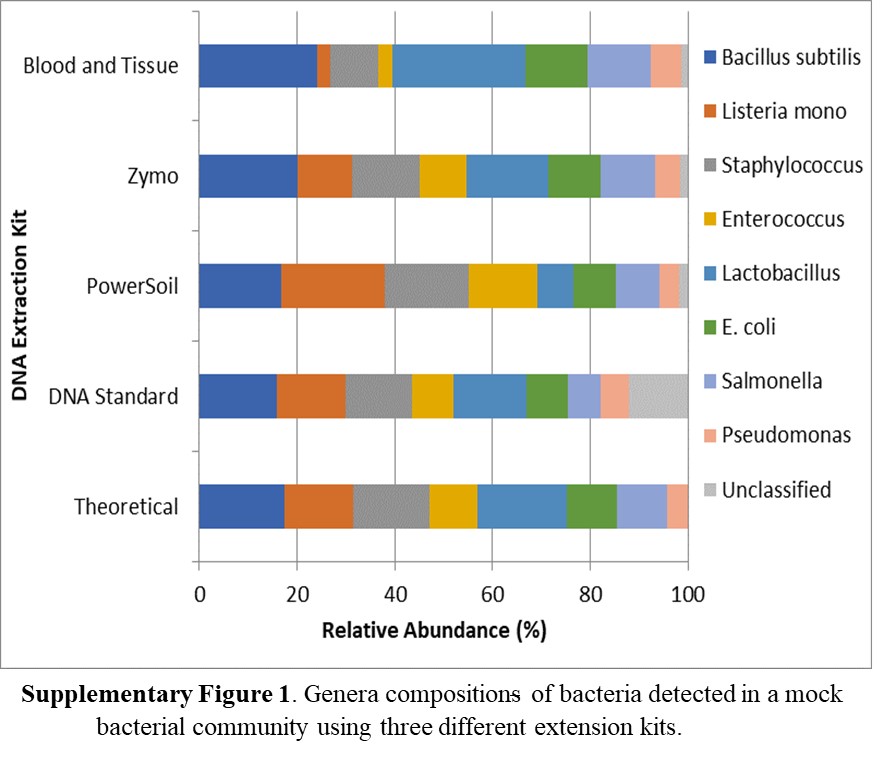

Supplement: Supplementary file 1 [file Image_1.jpeg]

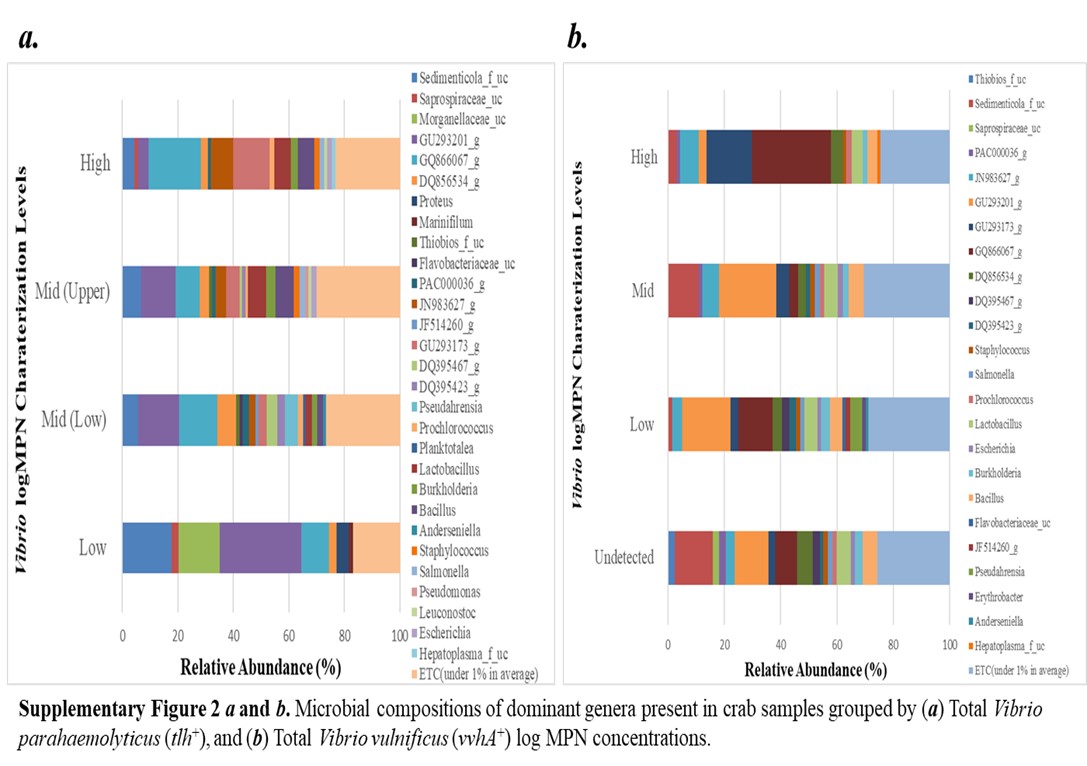

Supplement: Supplementary file 2 [file Image_2.jpeg]
